# Non-squamous Cancers of the Larynx

**DOI:** 10.1007/s11912-024-01535-4

**Published:** 2024-04-26

**Authors:** H. Bengu Cobanoglu, Erdal Rahman Koprucu

**Affiliations:** Faculty of Medicine, Department of Otorhinolaryngology Head & Neck Surgery, Karadeniz Technical University, Trabzon, Turkey

**Keywords:** Non-squamous, Larynx, Minor salivary gland, Sarcoma, Malignant melanoma

## Abstract

**Purpose of Review:**

Although non-squamous tumors of the larynx are really rare, they may not always be viewed from the same perspective in the multidisciplinary treatment approach once the diagnosis is made. In this review, non-squamous tumors of the larynx and current approaches in treatment will be discussed.

**Recent Findings:**

When the studies and meta-analyses presented in the last 5 years are evaluated, it is seen that these tumors usually show non-specific symptoms. Due to their submucosal location, the stage of the disease at the time of diagnosis is often advanced. In the literature, treatment may vary in these particular cases.

**Summary:**

The majority of non-squamous tumors of the larynx includes minor salivary gland tumors, neuroendocrine carcinomas, sarcomas, cartilage tumors, and malignant melanomas. Once treating a patient with these diagnoses, it should be kept in mind that the histopathological subtype is almost as important as the stage of the tumor.

## Introduction

Non-squamous cancers of the larynx account for less than 5% of all laryngeal cancers.

Non-squamous cancers have been discussed with different classifications in the literature. The most frequently used classifications are the World Health Organization (WHO) classification and the 8th edition classification of AJCC.

According to the WHO classification (5th edition), NSCCs of the larynx are divided into laryngeal cartilage tumors and others, whereas when we look at the AJCC classification, we see that the subgroups are slightly more diverse. These include minor salivary gland malignant tumors, neuroendocrine carcinomas, soft tissue tumors, malignant tumors of cartilage and bone, and hematolymphoid tumors.

In this review, rather than considering the main headings specified by the AJCC, the rarity of laryngeal.

- Minor salivary gland tumors
- Neuroendocrine carcinomas
- Sarcomas and cartilage tumors
- Malignant melanomas

Tumors, the fact that TNM staging is not very applicable, especially in sarcomas and the response to chemoradiotherapy is unpredictable, is among the obstacles in establishing a consensus on the management of NSCCs. In this review, the management of NSCCs will be discussed in light of recently published studies, cases, and meta-analyses in the literature.

## Minor Salivary Gland Tumors

The minor salivary glands of the larynx are located in the supraglottic area, such as the saccule, vestibular, and aryepiglottic folds, in the anterior part of the subglottic region and submucosally [1]. Salivary gland tumors of the larynx account for less than 1% of all laryngeal malignant tumors [2]. The majority of salivary gland malignant tumors of the larynx is mucoepidermoid carcinoma or adenoid cystic carcinoma [2]. Since these lesions originate from submucosal salivary glands, they are submucosally localized and may be asymptomatic until they reach a large size; therefore, they may be diagnosed late.

Malignant salivary gland tumors are not closely associated with smoking, unlike SCCs of the larynx [3, 4]. They occur equally in men and women. They are more common in the subglottic and supraglottic regions because the glands are more abundant in these areas. Symptoms are not different from the routine symptoms of laryngeal cancers. They may cause dysphonia, dysphagia or dyspnea depending on their location [2].

In malignant salivary gland tumors of the larynx, histological grade plays an important role in the survey [2]. In the literature, adenoid cystic carcinoma (ACC) (32–69%) and mucoepidermoid carcinoma (MEC) (15–35%) are the most common malignant minor salivary gland tumors [4, 5]. Adenocarcinoma, acinic cell carcinoma, and myoepithelial cell carcinoma are much rarer. There are a few case reports or case series in the literature [5, 6].

### Mucoepidermoid Carcinoma of the Larynx

MEC can be low or high grade. As mentioned above, the histologic grade is directly related to the survey. It is more common in men and the sixth decade of life. It is most commonly seen in the supraglottic area (60%) and specifically in the epiglottis. In addition, 30% are located in the glottic area and 10% in the subglottic or transglottic area—almost half of the patients present with a metastatic mass in the neck [7]. There is no consensus on the treatment of mucoepidermoid carcinoma of the larynx. Depending on the location of the lesion, partial or total laryngectomy is the primary treatment modality, but the issue of neck dissection in N0 patients is controversial. While some authors unequivocally recommend elective neck dissection for patients with high-grade MEC of the larynx [8, 9], others recommend elective neck dissection for all patients regardless of the grade of the tumor since the tumors of these patients are usually located supraglottically [10].

As with surgery, adjuvant treatment is also controversial. MECs are considered to be partially radiosensitive tumors, and therefore, RT is not the first choice for treatment. Post-operative radiotherapy is recommended in high-grade MEC [2]. While low-grade MEC has a survival rate of around 95%, this rate drops below 30% in high-grade MEC [11].

### Adenoid Cystic Carcinoma of the Larynx

Adenoid cystic carcinoma of the larynx is localized in the subglottic (57%), supraglottic (30%), and glottic (5.8%) areas, respectively [12]. There are tubular, cribriform, and solid subtypes in histologic appearance [2]. Since it is most commonly seen in the subglottic area, dyspnea and dysphonia are the major presenting symptoms. Sometimes, “pain” may be the main symptom due to the perineural invasion characteristic of ACC [13]. Although ACC is submucosal, the mucosa overlying the ACC mass in the larynx is usually ulcerated. As in classical salivary gland tumors, ACCs in the larynx show infiltrative growth patterns, perineural invasion, and a tendency to hematogenous dissemination [14]. The primary treatment modality is surgery. Cervical lymph node metastases are rare. However, late recurrences and distant metastases may be observed [15]. Distant metastases occur most commonly in the lung, liver, and bone. There is no consensus on whether the primary surgical treatment should be partial laryngectomy or total laryngectomy, which is a radical surgery.

Even in the largest series in the literature, the number of patients is limited. While some authors accept total laryngectomy as the gold standard treatment for ACCs in the larynx due to submucosal invasion and subglottic localization, some authors advocate partial laryngectomy, especially in supraglottic ACCs. In fact, in this disease, which may present with recurrences due to perineural invasion or distant metastases in the long term, oncologic principles have suggested preserving laryngeal function as much as possible [15, 16]. Elective neck dissection is recommended only in clinically or radiologically N-positive cases. Previously, ACCs were thought to be radioresistant, but now the general opinion is that ACCs are radiosensitive but not radiocurable. Chemotherapy (carboplatin/cisplatin ± paclitaxel) and radiotherapy can be used in primary and adjuvant larynx-preservation therapy, although this has only been tested in case-based articles [17, 18].

As a result, when we look at the mean survival times of laryngeal ACCs in the literature, the overall recurrence rate was 29% in the series of Lionello et al., while this rate was 17% in distant metastasis [15]. In another meta-analysis, the local recurrence rate was 13.3%, the regional metastasis rate was 7.8%, and the distant metastasis rate was 33.3% [19•].

In a cohort series of 15 patients published by Moukarbel et al., 5- and 10-year disease-specific survival rates were 69% and 49%, respectively [20].

## Neuroendocrine Carcinomas

Neuroendocrine carcinomas (NEC) account for less than 1% of all laryngeal tumors. Nevertheless, neuroendocrine tumors are the most common tumors of the larynx other than SCC. Neuroendocrine tumors are divided into two groups according to the tissue of origin: epithelial and neural [21]. Epithelial neuroendocrine tumors are called NECs, while those originating from the parasympathetic system are called paragangliomas [22]. Since paragangliomas are considered benign and only become malignant paragangliomas after metastatic lymph nodes, they will not be mentioned in this section. The answer to the question of which region of the head and neck NEC is most common is “the larynx.” In the larynx, it is most commonly observed in the supraglottic region [23, 24].

In 2017, the World Health Organization (WHO) divided NEC into four types: typical carcinoid (well differentiated), atypical carcinoid (moderately differentiated), small cell carcinoma (poorly differentiated), and large cell carcinoma (poorly differentiated type).

We have already mentioned that NEC is most commonly observed in the supraglottic region of the larynx [23]. Therefore, the presenting complaints of this group of patients include a feeling of catching in the throat, dysphagia, hoarseness, and a mass in the neck [25–28]. Sometimes, they may be asymptomatic without any symptoms and may be recognized during laryngoscopy or intubation for another reason [29]. Laryngeal NET is associated with cigarette smoking and is more common in the 6th–7th decade of males [30]. Histopathologic examination is very important in the diagnosis because the NEC subtype is determined, and treatment is decided accordingly.

### Typical Carcinoid Tumors

They are well-differentiated neuroendocrine carcinomas. Like other laryngeal NECs, they are associated with smoking and show male predominance [31]. Typical carcinoids rarely metastasize and are frequently located in the supraglottic region. Therefore, partial laryngectomies are the first choice in treatment. Lymph node metastasis is rare and usually does not require radio/chemotherapy [22, 32, 33].

### Atypical Carcinoid Tumors

It is the most common NEC subtype in the larynx. Like typical carcinoids, they are most commonly localized in the supraglottic region. They have both a locally aggressive course and a tendency to metastasize [28]. They may metastasize to lymph nodes, lungs, liver, pancreas, and breast. After the diagnosis is made, screening and subsequent treatment should be determined [29].

### Small Cell Carcinoid Tumors

It is the most lethal and aggressive subgroup. Distant metastasis usually develops at the time of admission. As in other subgroups, the main site of localization is the supraglottis. Since the patient usually has distant metastasis at the time of diagnosis, surgical treatment is not possible. Systemic chemo/radiotherapy is the main treatment. Unfortunately, patients die within 2–3 years [34]. In a series of 436 patients, Wan der Laan et al. reported a 5-year disease-free survey of 31% of patients treated with chemoradiation (CRT) [29].

### Large Cell Neuroendocrine Carcinomas

This group has the least information in the literature. Since it was not previously included in the definitions, its true incidence is unknown. Seventy percent of the patients are already stage 4 at the time of diagnosis. Therefore, they have lost their chance of surgery [29, 35, 36]. In a meta-analysis by Strojan et al., large and small cell NECs seen in the larynx were evaluated in terms of treatment and survey, and they could not show any significant variable other than the stage and histologic type of the disease in a multivariate model [28]. They reported that surgical treatments, systemic definitive 40–70 Gy, and post-operative 50–60-Gy radiotherapy were applied in patients. Although different chemotherapy agents have been tried over the years, the combination of platinum and etoposide is slightly superior to other agents [37].

## Sarcomas

Primary sarcomas of the larynx are rare and represent less than 1% of malignant neoplasms arising in this region [38, 39]. Chondrosarcoma is the most common subtype of primary sarcoma of the larynx and typically arises from multiple cartilage structures [40]. Other sarcomas are less common and occur mostly in the submucosal connective tissue [41].

Surgery constitutes the cornerstone of treatment in terms of therapeutic approach. Since most of these tumors are pedicled, their distant metastasis tendency is relatively lower, and they remain suitable for surgical resection for a longer period [42].

Sarcomas can have various morphologies, showing spindle, pleomorphic, anaplastic, small round, epithelioid, and giant cells. They can be confused with carcinomas, lymphomas, small cell carcinomas, mesothelioma, and melanomas. This situation poses diagnostic difficulties for inexperienced pathologists, especially in small laryngeal biopsies after the specimen is poorly obtained [43]. Some low-grade sarcomas may be confused with benign stromal neoplasms and tumor-like nodules. Being aware of the different types of sarcomas that may involve the larynx, paying attention to certain histomorphologic features, and performing ancillary histochemical studies help to reach the correct diagnosis [44].

### Soft Tissue Sarcomas

#### Synovial Sarcomas

The diagnosis of synovial sarcomas of the head and neck is particularly challenging because the tumor is rare in this region and resembles other more common tumors of the head and neck. Histologically, they are divided into two groups: monophasic and biphasic synovial sarcomas. Monophasic synovial sarcomas are characterized by the presence of only one cell component, spindle, or less frequently epithelioid, whereas biphasic synovial sarcomas are characterized by a mixture of spindle and epithelioid cells and are frequently organized in gland-like structures [45–49]. Biphasic synovial sarcomas need to be differentiated from carcinoma with partial sarcomatoid differentiation, whereas monophasic synovial sarcomas need to be differentiated from all spindle cell tumors, including hemangiopericytoma, malignant schwannoma and spindle cell carcinoma.

It may occur in all age groups, with young male patients being relatively more affected [46–50]. Symptoms may present as dysphagia, dyspnea, dysphonia, pain, and facial mass, depending on the area involved. In the literature, surgery has been reported as the first treatment option; total or partial surgery can be performed depending on the size of the lesion and the area involved. Since nodal metastasis is rare, elective dissection is not indicated in the absence of involvement. Radiotherapy has been shown to be effective in disease control; chemotherapy is controversial but may be useful in the presence of distant metastasis [45, 46, 48, 51–59]. In the literature, nodal metastasis is observed in 12% of synovial sarcomas, while distant metastasis is observed in 50% of cases [46, 47]. Five-year survival varies between 23.5 and 45%, while 10-year survival is between 11.2 and 30% [46, 60, 61].

#### Liposarcomas

Liposarcoma is one of the most common soft tissue malignant tumors and is frequently found in the lower extremities and retroperitoneum. Only approximately 5.6% of liposarcomas are found in the head and neck, and most tumors originate from the soft tissues of the neck. Laryngeal liposarcoma is extremely rare [62]. There are few cases of liposarcoma in the literature, and the cases that do exist have been described as tumors that are more common in men, are generally low-grade and easily excisable, and have a low tendency for nodal metastasis. Wide surgical excision has been recommended for treatment. Recurrence was generally associated with inadequate treatment, and nodal metastasis and distant metastasis were generally low [63].

#### Low-Grade Fibromyxoid Sarcoma

It is a sarcomatous malignant disease with a tendency for late recurrence and metastasis, which usually occurs in the 3rd and 4th decade and has a good survey despite the potential for metastasis [64–66]. The characteristic morphological features of low-grade fibromyxoid sarcoma consist of prominent curved vascular structures containing tumor cells and variable amounts of myxoid and fibrous areas. Collagen rosettes can be seen, and this is indicative of a more benign character [64, 65]. The features may be confused with spindle cell squamous cell carcinoma, and care should be taken in this respect [67].

#### Kaposi Sarcoma

Kaposi sarcoma (KS) is a multifocal, highly vascularized neoplasm with low-grade malignant potential that is thought to originate from endothelial cells of blood and lymphatic vessels [68, 69]. All Kaposi sarcoma subtypes are associated with human herpes virus 8 (HHV-8); the exact mechanism by which infected cells transform into KS is different for each subtype and is still under investigation [70, 71].

Kaposi sarcoma lesions usually appear as painless bluish-red or purple macules and eventually progress to hardened plaques and nodules [72, 73]. Histologically, KS is characterized by endothelial cells, fibroblasts, and spindle cells proliferating with scattered thin vascular clefts and extravasated erythrocytes [71, 74].

Before the AIDS epidemic, laryngeal manifestations were extremely rare, but literature reports have increased in recent years [74, 75]. Recognition of Kaposi sarcoma in the larynx is important because of the risk of airway obstruction [73, 76]. Treatment depends on the depth of invasion and involvement of surrounding structures. Most cases can be treated with endoscopic surgery and localized radiotherapy [74, 77]. More invasive treatment approaches may also be required depending on airway obstruction and the site of involvement. Alternatively, chemotherapy has been tried, especially in AIDS-related Kaposi sarcoma [78].

## Bone-Cartilage Sarcomas

### Chondrosarcoma

Primary chondrosarcoma of the larynx is a rare tumor and constitutes approximately 0.2% of all laryngeal malignancies. Despite its rarity, it is the third most common laryngeal malignancy after squamous cell carcinoma and adenocarcinoma [79]. As in epithelial tumors, it is frequently observed in males, and the majority of hospital admissions occur with hoarseness [40]. The etiology of laryngeal chondrosarcoma is unknown, but it has been suggested that it is caused by irregular ossification of the laryngeal cartilage [80, 81]. Laryngeal chondrosarcoma originates from the cricoid cartilage in most patients; they may also originate from the epiglottis, thyroid, arytenoid, and accessory cartilage [82].

Macroscopically, it appears as a smooth, lobulated mass [82]. Histologically, it has hyperchromatic nuclei with little stroma [39]. The 5-year survival rate in chondrosarcoma is high; two studies reported 5-year survival rates of 79.4% and 88.6%, respectively [2, 8]. Although the rate of metastasis is low, they are locally aggressive tumors [83]. While low-grade tumors can be successfully treated with new surgical techniques such as CO_2_ lasers, high-grade and recurrent tumors require more aggressive treatment approaches, such as partial or total laryngectomy [82]. Therefore, despite the favorable prognosis of chondrosarcoma, patients with recurrent disease may lose the ability to phonate and swallow, significantly reducing quality of life.

Although the main treatment of chondrosarcoma is surgery, the role of radiotherapy is controversial, and chemotherapy is not considered beneficial [84]. However, radiotherapy has started to be advocated due to the reports of complete remission with radiotherapy in chondrosarcomas in different regions reported in the literature [84].

When chondrosarcomas of other parts of the body are compared with laryngeal chondrosarcomas, laryngeal chondrosarcomas tend to be low-grade and less aggressive [82, 85]. Even in high-grade histologies, the likelihood of metastasis is relatively low [86]. Histopathologic grades are classified from grade I to III. The most common presentation is grade I. While traditional subtypes constitute 97.9% of laryngeal sarcoma histopathologically, rare pathologic variations include clear cell, myxoid, and dedifferentiated subtypes [87].

Since it is a rare tumor, information about laryngeal chondrosarcomas is generally obtained from case series and reviews of these series. In a review of radiologic features of laryngeal chondrosarcomas, 74.5% were located in cricoid cartilage, 12.7% in cricoid and thyroid cartilage, 7.3% in thyroid cartilage, and 5.5% in arytenoid cartilage. Well-defined margins were demonstrated in all lesions in the series examined. On CT, cortical defect/enhancement was observed in 98%, internal low density in 89.6%, calcification in 95.8%, and homogeneous and low-contrast uptake on contrast-enhanced CT in 85.3%. MRI showed a high signal on T2-weighted imaging, a low signal on T1-weighted imaging, and heterogeneous and mild contrast enhancement on postcontrast T1-weighted imaging in all cases. Pathologic grading and radiologic findings were not found to be significant in this study [88].

Treatment of laryngeal chondrosarcomas is mainly determined by the size and localization of the tumor, and histological grading is used as an auxiliary evaluation tool [10]. Conservative, function-preserving surgery is the first preferred treatment method, but radical methods may also be preferred depending on the size of the tumor [40, 81, 86]. Although there is a consensus for a conservative approach for tumors in the thyroid cartilage, arytenoids, and epiglottis, the treatment of chondrosarcomas in the cricoid region is controversial because they are easily resectable tumors [89]. Total laryngectomy is preferred in cases of laryngeal dysfunction after excision in which the cricoid cartilage is usually involved [90]. The surgical tendency is to perform total laryngectomy when laryngeal chondrosarcoma exceeds half of the cricoid cartilage [91].

### Osteosarcomas

Although it is the most common malignant neoplasm of the bony skeleton, it is one of the rarest mesenchymal neoplasms of the larynx. It constitutes 1% of head and neck cancers. It is most commonly seen in the mandible 45–56% and in the maxilla 32–40% [92, 93]. In one study, a 5-year survival rate of 59.7% was found [94]. The most common sarcoma encountered in the larynx is chondrosarcoma; therefore, even specialists in academic centers working on a reference basis encounter a single case in their careers [95].

Among previously reported cases, the most common complaints were hoarseness and dyspnea. Dysphasia, odynophagia, and acute airway obstruction were also observed in some patients. In these few reported cases, there was no evidence of a correlation with alcohol intake or smoking [96].

It is remarkable that the morphologic features of sarcomas typically represent a heterogeneous variation. For this reason, biopsies do not contain sufficient material, which causes difficulties in differential diagnosis. Unlike chondrasarcomas, the diagnosis of malignancy by pathologists is clear because it is a high-grade malignancy.

Microscopically, the tumor consists of spindle-shaped mesenchymal cells with a clearly malignant appearance associated with osteoid and immature neoplastic bone formation. They show multiple mitoses, and the nuclei show significant hyperchromasia and pleomorphism. Venous invasions can be detected [95].

The definitive diagnosis of osteosarcoma depends on the identification of osteoid production by malignant cells in the biopsy specimen. The differential diagnosis includes spindle cell squamous carcinoma with osteoid differentiation, chondrosarcoma, malignant fibrous histiocytoma, fibrosarcoma, and myositis ossificans affecting the larynx. Spindle cell squamous carcinoma may contain benign and rarely malignant osteoid areas [97].

Typically, wide local resection with clean margins, such as total laryngectomy, is preferred to achieve long-term survival. Since osteosarcomas are considered radioresistant, neoadjuvant radiotherapy after surgery should be evaluated individually. Chemotherapy may contribute favorably to the prognosis in patients with extensive lesions [95].

## Malignant Melanoma

When a diagnosis of malignant melanoma of the larynx is made, it is necessary to determine whether it is a primary laryngeal mucosal malignant melanoma originating from melanocytes of the larynx or metastasis of cutaneous malignant melanoma. The incidence of primary malignant melanoma in the larynx is quite low [98]. In the larynx, melanocytes are found in the basal cell layer of the epithelium, submucosa stroma, or minor salivary glands in the submucosa [99]. Therefore, malignant melanomas are most commonly located in the supraglottic region. On laryngeal examination, these tumors may appear as brown, gray or purple, nodular, stalked, or polypoid masses [100]. Immunohistochemical staining is the gold standard for diagnosis. The patient should be investigated and imaged in detail in terms of primary lesions and metastases. Cervical and distant metastases are present in many patients at the time of diagnosis. The optimal treatment is complete surgical excision, but this is not possible in most patients due to distant metastases. Adjuvant radiotherapy improves locoregional control but does not affect survival [101–103]. In a systematic review by Fernandez et al., overall survival was reported as 46% for 2 years and 12% for 5 years, while disease-free survival was 41% for 2 years and 10% for 5 years. The most common cause of death is distant metastasis (55% of cases) [104].

## Conclusion

The presence of distant metastasis should be investigated in patients with non-squamous laryngeal cancer which is usually diagnosed at a late stage. Surgical treatment should be prioritized in non-metastatic cases but the subsequent treatment of these patients should be handled by an experienced multidisciplinary team. It should be kept in mind that the histopathological subtype is almost as important as the stage of the tumor.
